# Prevalence and Molecular Characterization of Extended-Spectrum β-Lactamases and AmpC β-lactamase-Producing *Enterobacteriaceae* among Human, Cattle, and Poultry

**DOI:** 10.3390/pathogens11080852

**Published:** 2022-07-28

**Authors:** Mohamed A. Nossair, Fatma A. Abd El Baqy, Mohammad S. Y. Rizk, Haitham Elaadli, Alaa M. Mansour, Ayman H. Abd El-Aziz, Adil Alkhedaide, Mohamed Mohamed Soliman, Hazem Ramadan, Mustafa Shukry, Sabah I. Shaaban

**Affiliations:** 1 Department of Animal Hygiene and Zoonoses, Faculty of Veterinary Medicine, Alexandria University, Alexandria 21544, Egypt; mohammadnossair@alexu.edu.eg (M.A.N.); fattmaahmed930@gmail.com (F.A.A.E.B.); haytham.kamal@alexu.edu.eg (H.E.); alaa.m.mansour@alexu.edu.eg (A.M.M.); 2 Department of Microbiology, Animal Health Research Institute, Damanhur 22511, Egypt; mohamedrizk@gmail.com; 3 Animal Husbandry and Animal Wealth Development Department, Faculty of Veterinary Medicine, Damanhour University, Damanhour 22511, Egypt; ayman.sadaka@vetmed.dmu.edu.eg; 4 Clinical Laboratory Sciences Department, Turabah University College, Taif University, Taif 21995, Saudi Arabia; a.khedaide@tu.edu.sa (A.A.); mmsoliman@tu.edu.sa (M.M.S.); 5 Hygiene and Zoonoses Department, Faculty of Veterinary Medicine, Mansoura University, Mansoura 35516, Egypt; hazem_hassan@mans.edu.eg; 6 Department of Physiology, Faculty of Veterinary Medicine, Kafrelsheikh University, Kafrelsheikh 33516, Egypt; 7 Department of Animal Hygiene and Zoonoses, Faculty of Veterinary Medicine, Damanhour University, Damanhour 22511, Egypt; sabah_ibrahim@vetmed.dmu.edu.eg

**Keywords:** ESBL, AmpC, *Enterobacteriaceae*, Egypt

## Abstract

Extended-spectrum beta-lactamase (ESBL)-producing *Enterobacteriaceae* are a universal public health alarm frequently identified among humans, animals, and poultry. Livestock and poultry production are a possible source of multidrug-resistant microorganisms, including ESBL-producing *Enterobacteriaceae*, which confer antimicrobial resistance to different β-lactam antimicrobial agents. From January to May 2020, a cross-sectional study was carried out in three dairy cattle farms and four poultry farms in different districts of northern Egypt to assess the prevalence of ESBLs, AmpC beta-lactamase-producing *E. coli* and *Klebsiella* in livestock, poultry, and human contacts, and to investigate the genetic relatedness of the recovered isolates. In total, 140 samples were collected, including human fecal samples (n = 20) of workers with intimate livestock contact, cattle rectal swabs (n = 34), milk (n = 14), milking machine swabs (n = 8), rations (n = 2), and water (n = 2) from different cattle farms, as well as cloacal swabs (n = 45), rations (n = 5), water (n = 5) and litter (n = 5) from poultry farms. The specimens were investigated for ESBL-producing *E. coli* and *Klebsiella* using HiCrome ESBL media agar. The agar disk diffusion method characterized the isolated strains for their phenotypic antimicrobial susceptibility. The prevalence of ESBL-producing *Enterobacteriaceae* was 30.0%, 20.0%, and 25.0% in humans, cattle, and poultry, respectively. Further genotypic characterization was performed using conventional and multiplex PCR assays for the molecular identification of ESBL and AmpC genes. The majority of the ESBL-producing *Enterobacteriaceae* showed a multi-drug resistant phenotype. Additionally, *bla***_SHV_** was the predominant ESBL genotype (n = 31; 93.94%), and was mainly identified in humans (n = 6), cattle (n = 11), and poultry (14); its existence in various reservoirs is a concern, and highlights the necessity of the development of definite control strategies to limit the abuse of antimicrobial agents.

## 1. Introduction

Antimicrobial resistance (AMR) has become an evolving hazard to public health because of unnecessary antimicrobial exposure in human and veterinary medicine, predominantly in unindustrialized countries like Egypt [1]. Antibiotics are utilized in livestock at a global level of 63,000 tons, and this number is expected to rise to 105,000 tons in 2030 [2]. These acts contribute to the extensive proliferations of AMR pathogens in humans, cattle, and the environment, resulting in a more extended hospitalization for patients, a financial burden on the community, and lethal effects [2]. 

Diseases caused by drug-resistant *Enterobacteriaceae* have become a serious threat worldwide. In particular, resistance to beta-lactam antibiotics is a significant concern in the medical and veterinary sectors [3]. The principal resistance mechanism is the production of the extended-spectrum lactamases (ESBLs), which can hydrolyze penicillin, broad-spectrum cephalosporins, and monobactams produced from *TEM*, SHV, and CTX-M type enzymes, which are inhibited by clavulanic acid and other B-lactamase antagonists [4]. ESBLs are often found on plasmids that can be simply shifted across bacterial species and from one strain to another [5]. The occurrence of ESBLs is rising in various places of the world. Furthermore, AmpC-type β- lactamases are frequently isolated from many ESBL-producing *Enterobacteriaceae*. AmpC β- lactamases, in contrast to ESBLs, hydrolyze extended-spectrum cephalosporin but are not blocked by clavulanic acid or other β-lactamase inhibitors, and are resistant to cephamycins such as cefoxitin and cefotetan [4]. 

The most commonly found ESBL-producing *Enterobacteriaceae* are *Klebsiella* and *E. coli* spp, which frequently cause healthcare problems and society-acquired infections [6]. Clinically related ESBL-producing *Enterobacteriaceae* are not limited to humans, and have also been described in food-producing animals, poultry, and the environment [7]. Unfortunately, there is no legislation regulating antimicrobial agents’ use in Egypt. Some antimicrobials, such as beta-lactams, quinolones, and tetracycline, are still employed for purposes other than treatment [8]. The irrational usage of antimicrobials causes the rapid emergence of multi-drug-resistant species of *Enterobacteriaceae* in cattle and poultry. This plays a significant part in disseminating bacteria which are resistant to antibiotics, which make their way up the food chain to humans [9,10,11]. Furthermore, research has revealed that these resistant bacteria are circulated via the food production chain or through immediate contact between humans and cattle [12,13].

In Egypt, several reports have been created on ESBL-producing *Enterobacteriaceae* in chickens, cattle, and humans [14,15]. However, little is known about AmpC β-lactamase-producing *Enterobacteriaceae* in cattle or poultry. This work aimed to determine the existence of ESBL (*blaSHV*, *bla_TEM_*, and *bla_CTX-M_*) and *AmpC* β-lactamase-producing *E. coli* and *Klebsiella* spp in humans, cattle, and poultry in Egypt, and to investigate the genetic similarity of isolates from the various hosts.

## 2. Results

Of the 140 samples, 33 (23.57%) *E. coli* and *Klebsiella* samples were recovered on HiCrome ESBL media agar, distributed as six (30%) isolates from humans, 12 (20%) from cattle, and 15 (25%) from poultry (Table 1). All 33 *E. coli* and *Klebsiella* isolates were tested for their antimicrobial susceptibility. Of the examined 33 isolates, 28 (84.85%) were resistant to cefotaxime and ceftazidime, seven (21.21%) were resistant to amoxicillin/clavulanic acid, 20 (60.61%) were resistant to levofloxacin, three (9.1%) were resistant to imipenem, and 23 (69.7%) were resistant to cefepime (Table 2). 

**Figure 1:**
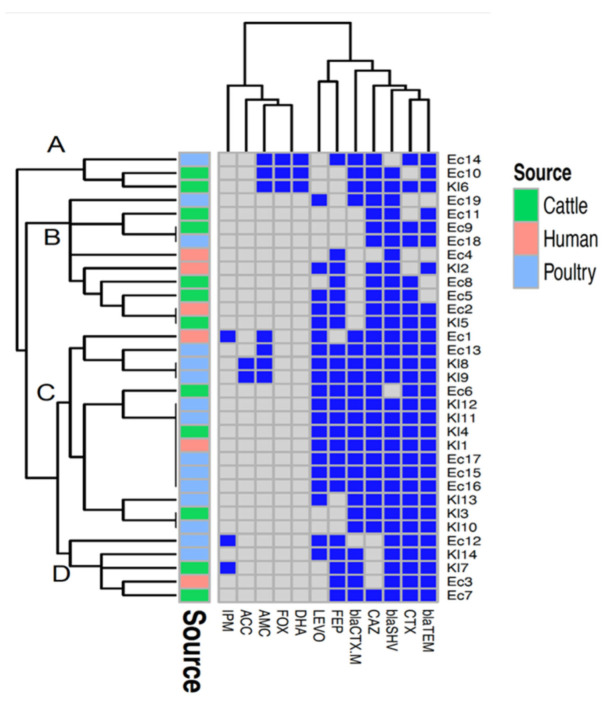
A heatmap supported by a dendrogram showing the distribution of the antimicrobial resistance genes and resistance phenotypes among the examined *Escherichia coli* and *Klebsiella* isolates from humans, cattle, and poultry. Dark blue squares indicate resistance genes and phenotypic resistance; gray squares indicate absent genes and phenotypic susceptibility. Four clusters (A–D) are indicated in the figure.

The 33 suspected ESBL-producing isolates were then subjected to the double-disk synergy test method (DDST), which confirmed 30 isolates as ESBL-producers and three as non-ESBL-producers. The PCR screening of the isolates to β -lactam resistance genes revealed that *bla*_SHV_ (31/33, 93.94%) was the predominant gene, followed by *bla*_TEM_ (29/33, 87.88%) and *bla*_CTX-M_ (23/33, 69.7%). The multiplex PCR assay detected 15.15% AmpC genes (n = 5) among the *E. coli* and *Klebsiella* isolates (Table 3). Based on phenotypic and genotypic resistance profiles, the generated heatmap clustered the isolates into four groups (A–D). Group C was the largest, comprising 15 isolates sourced from the three hosts (human, cattle, and poultry). Identical profiles of antimicrobial resistances were found among three pairs of isolates from different hosts: two pairs from cattle and poultry (Ec9 and Ec18, and Kl3 and Kl10), and one pair from humans and cattle (Ec2 and Kl5). Seven isolates (five isolates from poultry: Kl12, Kl11, Ec17, Ec15, Ec16; one isolate each from cattle Kl4 and human Kl1) also had identical phenotypic and genotypic resistance profiles. Another pair of isolates belonging to poultry (Kl8 and Kl9) showed identical profiles of antimicrobial resistances (Figure 1).

**Figure 2:**
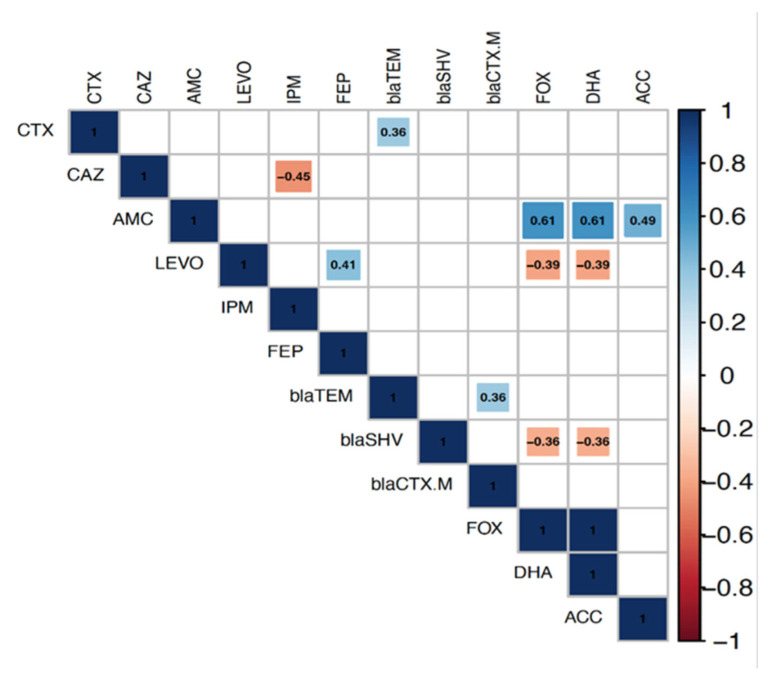
Correlation analysis determines the associations between resistance genes and antimicrobial resistance phenotypes among *Escherichia coli* and *Klebsiella* isolates from humans, cattle, and poultry. The blue and red colors of the boxes indicate positive and negative correlations, respectively. The strength of the color corresponds to the numerical value of the correlation coefficient (*r*). Significance was calculated at *p* < 0.05, and boxes with non-significant correlations were left blank.

The correlation analysis determined the relationship between the antimicrobial resistance genes and phenotypic antimicrobial resistances among the isolates. Significant positive correlations were observed for the co-occurrence of resistance genes and phenotypic resistance to their corresponding antimicrobials. The *AmpC* genes *bla*_FOX_, *bla*_DHA_, and *bla*_ACC_ were significantly and positively correlated with AMC, and the correlation coefficients (r) were 0.61, 0.61, and 0.49, respectively. Furthermore, the *bla*_TEM_ gene showed a significant positive association with CTX (r = 0.36). On the other hand, significant negative correlations were found between resistance genes and antimicrobials other than the corresponding ones, i.e., *bla*_FOX_ and *bla*_DHA_ genes with LEVO (r= −0.39). Concerning the gene/gene relationship, a significant positive correlation was observed for the co-existence of *bla*_TEM_ and *bla*_CTX-M_ genes (r = 0.36). In contrast, significant negative correlations were found between *AmpC* genes, *bla*_FOX_ and *bla*_DHA_, and the *bla*_SHV_ gene(r= −36) (Figure 2).

Six representative CTX-M amplified sequences were chosen from *E. coli* and *Klebsiella* positive isolates from each host species for sequence analysis with other ESBL-producing *Enterobacteriaceae* strains. The selected samples were named KHF, KCF, KPF, EHF, ECF, and EPF. The sequencing results were submitted to GenBank (accession no. MZ461491, MZ461492, MZ461493, MZ461494, MZ461495 and MZ461496), as shown in Table 4. CTX-M 15 was the most prevalent enzyme in the sequenced isolates.

## 3. Discussion

Resistance to antimicrobials is a significant public health concern because the resistant bacteria and their movable genetic elements (plasmids, transposons, and integrons) disseminate among humans, animals, and the environment with consequential worldwide spreading [16,17]. Specifically in developing countries, the appearance and re-emergence of diseases caused by antibiotic-resistant pathogens are primarily due to ineffective hygienic strategies, immediate contact with animals, and unhygienic food handling and consumption practices [18]. For these reasons, a universal effort called One-Health was established to create an integrated attitude to work in a supportable way, in order to focus on the surveillance of antimicrobial-resistant organisms at the human–animal interface. 

In particular, the resistance of *Enterobacteriaceae* to extended-spectrum cephalosporins is a global issue [19], and is primarily caused by ESBL production. This problem is exacerbated by the production of extra-beta lactamases (AmpC). Besides this, the presence of the AmpC genes is regularly linked with multiple-drug resistance [20]. The global dissemination of ESBL-producing strains of *Enterobacteriaceae* provides a high significance to the study of the co-existence of these strains in humans, cattle, and poultry in Egypt.

Our study showed that the overall percentage of ESBL-producing bacteria was (23.57%) in our study. This finding was somewhat in line with that recorded by Egbule and collaborators [21], who noticed that the occurrence of ESBL *E. coli* isolated from humans, cattle, and poultry in Pakistan was 19.3 %, and [22] reported that the prevalence of ESBL-producing *Enterobacteriaceae* ranged from 11 to 72% in humans, was 0% in cattle, and ranged from 11 to 36% in poultry. On the contrary, these results differed from those [22] which found that the overall prevalence of ESBL-producing *Enterobacteriaceae* was 86.7% in Germany.

The percentage of ESBL-producing bacteria in human samples was 30%, which was higher than that found in [23], which reported that the percentage of ESBL producers was 6.8% in farm workers. In summary, this carriage rate of ESBL producers could be attributed to factors such as the low socioeconomic status of farm workers, the lack of hygienic practices, and close contact between humans and animals. Furthermore, selective antibiotic pressure caused by the extensive use of third-generation cephalosporins [24] may be the reason for this human carriage rate in our study in Egypt.

Our findings revealed a high percentage of fecal carriage of ESBL-positive isolates recovered from cattle. The current results (20.6%) were higher than a report from Burgundy in France in 2012, which revealed only a low occurrence—about 5%—of ESBL-producing bacteria in fecal specimens from the examined farms [25]. In contrast, the results were lower than those documented by [8], who found that the level of ESBL-producing *E-coli* was 42.8% in rectal swabs from apparently healthy cattle in Egypt. In addition, two records from Germany described the frequency of ESBL-producing bacteria in apparently healthy animals [12,13]. Schmid et al. [13] collected 598 specimens that contained 196 ESBL-producing *E. coli* (32.8%). Moreover, Dahms et al. [12] examined various farms for ESBL-producing bacteria and recorded that 70.6% of the tested farms were positive for ESBL-producing bacteria. This percentage of ESBL-producing *E. coli* in apparently healthy animals shows the considerable zoonotic risk for people who come in close contact with livestock.

The presented data in Table 3 showed that the *bla*_SHV_ gene was the most predominant in 31 isolates (93.94%), and this result was contrary to the results recorded by [26], who found that the *bla*_SHV_ gene was the least-often observed (6.52%). The high presence of *bla*_SHV_ may be the reason for the high phenotypic resistance to cephalosporin.

It was noticed that 21.21% of the isolates were phenotypically related to ESBL molecular class C. They showed more activity on extended-spectrum cephalosporins with the usual resistance to clavulanic acid (beta-lactamase inhibitor). This expression could be attributed to numerous enzymes such as AmpC enzymes (*bla*_ACT-1_, *bla*_CMY-2_, *bla*_FOX-1_, *bla*MIR-1, *bla*_GCI_, and *bla*_CMY-37_) or class A ESBL enzymes (*bla*_TEM-50_, *bla*_TEM-30_, and *bla*_SHV-10_) [27]. The five isolates (two from cattle and three from poultry) harbored *AmpC* genes; *bla*_FOX_, *bla*_DHA_, and *bla*_ACC_ were also found with phenotypic resistance to amoxicillin/clavulanic acid, besides other cephalosporins (cefotaxime, ceftazidime, and cefepime). A substantial contribution to this high spread of ESBL/AmpC-producing strains is the extensive usage of third generation cephalosporins, particularly on farms, keeping healthy and diseased livestock together nearby, and poor sanitation.

The high fecal carriage rate of ESBL-producing bacteria in cattle raises the question of how to dispose of highly contaminated feces. Feces are well known for their use as fertilizers in agriculture. Multidrug-resistant microorganisms could enter the food chain in this way, either directly through meat consumption or indirectly through cattle feeding on fertilized fields, posing a serious threat to the environment and the human population. Carbapenem-resistant isolates were also phenotypically retrieved. It is unknown whether carbapenems are used in the veterinary field, suggesting that this resistance may be transmitted through gene transfer from the human to the cattle population. This finding was compatible with [28], who detected a resistance phenotype against imipenem (42%), ertapenem (35%), doripenem (30%), and meropenem (28%) in cattle fecal samples. The recognition of the carbapenem-resistant phenotype and the risk of multiple drug-resistant pathogens ending in food (e.g., milk and dairy products) increase the dangerous threats to human health. The most frequently responsible enzymes for carbapenem resistance are molecular class A (KPC-2, IMI-1, SME-1) followed by class B metallo-β-lactamases (IMP-1, VIM-1, CcrA, IND-1, L1, CAU-1, GOB-1, FEZ-1, CphA, Sfh-1), along with class D (OXA-23, OXA-48) [27]. 

Poultry is one of the most broadly consumed foods worldwide, and several antimicrobials are used throughout poultry production in many countries. The threat of increasing antimicrobial-resistant organisms in the poultry environment may cause danger to human lives [29]. In our report (17.78%), ESBL producers were predominantly detected from cloacal swabs, and this result followed [30] the identified 13.7% of ESBL-positive isolates from poultry. Our findings suggested that antibiotics are extensively used in broiler production in Egypt, and have also been given as growth promoters rather than treatment. 

The findings of the heatmap analysis and hierarchical clustering (Figure 2) based on genotypic and phenotypic resistance profiles revealed the clustering of isolates belonging to different hosts. Besides this, pairs of isolates sourced from the three hosts (human, cattle, and poultry) showed identical genotypic and phenotypic resistance profiles. This was not surprising, as the shared resistance genes in these isolates—including *bla*_TEM_, *bla*_SHV,_ and *bla*_CTX-M_—are plasmid-mediated genes that can be transferred between human and animal-derived isolates [31]. 

**Figure 3:**
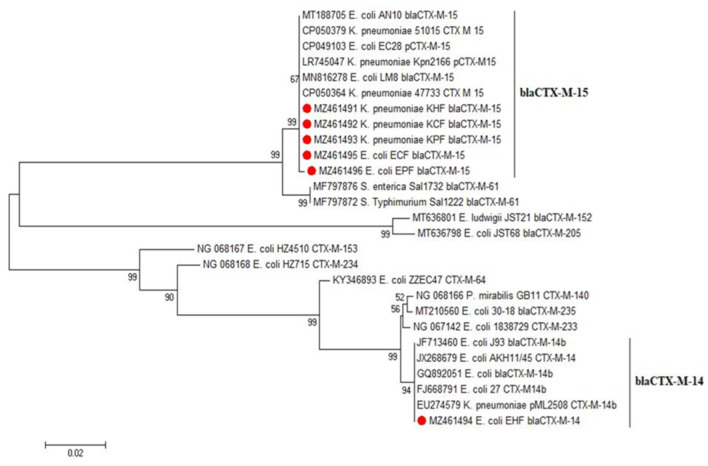
Phylogenetic tree of ESBL-producing *Enterobacteriaceae* based on partial nucleotide sequences of the CTX-M gene. N.B.: The isolates from this study are indicated by a red circle.

The data shown in Table 4 represent the samples selected for sequence analysis based on the CTX-M gene. It was found that all of the samples were genetically related to CTX-M-15, while only one *E. coli* sample isolated from humans was associated with CTX-M-14. This high rate of CTX-M-15-containing isolates is a potential risk. This genotype is evolving worldwide, and is linked with multidrug-resistant pathogens, causing community and hospital-acquired infections [32,33]. This high detection rate of bla_CTX-M-15_–containing isolates agreed with the preceding records on *bla*_CTX-M-15_ being the most frequent ESBL in the Middle East and North Africa [34]. This high prevalence of the *bla*_CTX-M-15_ allele might owe itself to the potent ability of its gene products (CTX-M-15 and its variants) to break the structure of ceftazidime, cefotaxime and aztreonam, which possibly provides the bacteria with a selective advantage, especially when various antibiotics are concurrently or successively prescribed. This high genetic matching can suggest the zoonotic transmission of strains between human and animal populations. This finding was in harmony with [35], who identified the same type of CTX-M (*bla*CTX-M-15) among ESBL-E in Egypt, and [36], who detected blaCTX-M-14, -15, -27, and blaCTX-M-55 variants in various animal species; their presence could be an indication of transmission between humans and animals because these strains have been associated with the global spread of blaCTX-M in human clinical isolates [7]. Figure 3 displays the phylogenetic tree of the current CTX-M type class A β- lactamase and other published CTX-M sequences showing high genetic similarities (99% identity), which may explain the possibility of the exchange of this resistance gene between species in some Egyptian districts. 

## 4. Material and Methods

### 4.1. Ethical Declaration

This research was performed following the Ethics of the Alexandria University Institutional Animal Care and Use Committee guidelines (ALEXU-IACUC, 3312020, Egypt). There was a comprehensive discussion with each worker before sample collection, and each signed a declaration of consent to participate in this research study. The ethics statement was not required regarding the cattle and poultry specimens because the swabs were collected by a non-invasive method, and no experiments were conducted for this surveillance study.

### 4.2. Sample Collection

**Figure 4:**
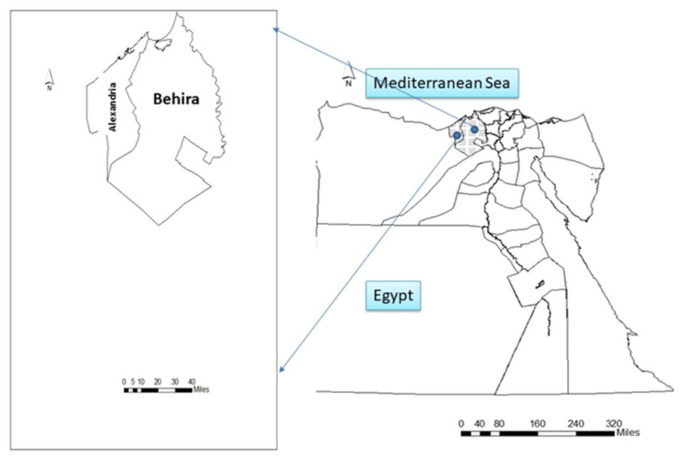
Map of Egypt showing the study areas.

Samples were collected from various humans, cattle, and poultry in several districts of the Behera and Alexandria Governorates in Egypt from January to May 2020 (see Figure 4). One hundred and forty specimens were gathered from human contact workers, dairy cattle, and broiler chickens, including human fecal samples (n = 20) from the workers, rectal swabs (n = 34), milk (n = 14), milking machine swabs (n = 8), rations (n = 2) and water (n = 2) from the cattle farms. Furthermore, cloacal swabs (n = 45), rations (n = 5), water (n = 5) and litter (n = 5) were taken from poultry farms. All specimens were taken and transferred in an ice box to the Animal Hygiene and Zoonoses Department laboratory, Faculty of Veterinary Medicine, Alexandria University for bacteriological analysis. 

### 4.3. Isolation and Identification of ESBL- Producing Enterobacteriaceae

The inoculated plates were incubated under aerobic conditions at 37 °C for 24–48 h. The specimens (n = 140) were primarily streaked on MacConkey agar (Oxoid, Hampshire, UK). The positive isolates were subcultured on Eosin Methylene blue agar (Oxoid, Hampshire, UK); then, the positive isolates were finally cultured in HiCrome ESBL media agar (Himedia), on which a presumptive ESBL producer produces bluish-green colonies. Simmons Citrate Agar was used to differentiate between *E. coli* and *Klebsiella*. The colonies were picked and preserved in aliquots of nutrient broth with glycerol for further microbiological examination [37]. 

### 4.4. Testing for Susceptibility to Antimicrobials 

Commercially available antibiotic disks (HiMedia) were used for antimicrobial susceptibility testing. According to the Clinical Laboratory Standards Institute (CLSI) guidelines, the disc diffusion method was used to assess the antimicrobial susceptibility. Samples were inoculated onto Muller–Hinton agar (MHA) (Oxoid). The following antibiotic disks were used: ceftazidime (30 μg), amoxicillin/clavulanic acid (30 μg), cefotaxime (30 μg), levofloxacin (30 μg), cefepime (30 μg), and imipenem (30 μg). After 24 hours of incubation at 37 °C, the diameter of the inhibitory zone was measured [38]. 

### 4.5. Phenotypic Confirmation of ESBL

The phenotypic verification of the ESBL-producing Enterobacteriaceae was carried out with a double-disk synergy test, as previously reported [39]. In brief, each isolate was inoculated on the MHA plate. Then, an amoxicillin/clavulanic acid disk (AMC, 20/10 μg) was placed 25 mm from the ceftazidime (CAZ) (30 µg) and cefotaxime (CTX) (30 µg) disks. After incubation, the increase in the CAZ inhibition zone or CTX disks toward the AMC disk (keyhole shape) was recorded as positive ESBL production.

### 4.6. DNA Isolation and Detection of ESBL and AmpC Type β-lactamase Genes 

Bacterial isolates were cultivated in nutrient broth at 37 °C for 12 h; the bacterial cultures were mixed with distilled water, and the suspension was centrifuged at 10.000× *g* for 5 min. The bacterial pellet was resuspended in distilled water and boiled in a water bath for 10 minutes, then centrifuged at 10.0000× *g* for 5 min. The DNA extraction was performed using the boiled cell method [17]. Finally, the supernatant was collected and used as a DNA template for PCR.

Conventional and multiplex PCR was performed using the primer sets in Table 5. The reaction of the conventional PCR was conducted in a total volume of 10μL in order to detect bla _SHV_, bla*_TEM_*, and bla_CTX-M_ genes. In contrast, the reaction of multiplex PCR was performed in a total volume of 40μl in order to detect AmpC genes (bla_MOX_, bla_CIT_, bla_DHA_, bla_EBC_, bla_FOX_, and bla_ACC_), as shown in Table 6. The PCR products were analyzed on ethidium-bromide-stained 1.5% agarose gel, and the bands were visualized under a UV transilluminator.

### 4.7. DNA Sequencing

Based on CTX-M gene product, six isolates (3 *klebsiella* and 3 *E-coli*) were selected from the three hosts for sequence analysis. The purified extracted PCR products were sequenced in the forward and/ or reverse directions on an Applied Biosystems 3130 automated DNA Sequencer (ABI, 3130, USA) using a ready reaction Bigdye Terminator V3.1 cycle sequencing kit. (Perkin-Elmer/Applied Biosystems, Foster City, CA, USA, Cat. No. 4336817). 

### 4.8. Phylogenetic Analysis of the Sequenced Genes

A comparative analysis of the sequences was performed using the CLUSTAL W multiple-sequence alignment program, version 1.83 of the MegAlign module of Lasergene DNA Star software Pairwise [40]. Phylogenetic analyses were conducted using maximum likelihood, neighbor-joining, and maximum parsimony in MEGA6 [41].

### 4.9. Statistical Analysis

The data were collected and processed. The statistical analysis results are presented in the tables and figures using SPSS statistical software, version 16.0. A correlation analysis was performed to determine the association between the antimicrobial resistance genes and phenotypic antimicrobial resistances among the isolates. The resistance phenotypes and genotypes results were converted into binary data (0/1); the absence of resistance genes or susceptibility to antimicrobials had scores of 0, while the presence of a resistance gene or resistance to antimicrobials received scores of 1. Binary data were imported into R software (version 3.6.1; https://www.rproject.org, accessed on 4 July 2022); with the “corrplot” package, the correlations were determined at a significance of *p* < 0.05 using the functions “cor” and “cor.mtest”. A heatmap with hierarchical clustering was also generated using the R packages “heatmap” and “RColorBrewer”, in order to cluster the examined isolates based on their phenotypic and genotypic resistance profiles.

**Table 5:** Oligonucleotide primer sequences of the PCR assay.

Target Genes	Nucleotide Sequence (5'to 3')	Amplicon Size (bp)	Reference
Bla _MOX-1_, Bla _MOX-2_, bla _CMY-1_, bla _CMY-8 TO_ bla _CMY-11_	GCTGCTCAAGGAGCACAGGAT CACATGACATA GGTGTGGTGC	520	[42]
Bla _LAT-1_ TO Bla _LAT-4_, Bla _CMY-2_ TO Bla _CMY-7_, Bla _BIL-1_	TGGCCAGA CTGACAGGCAAA TTTCTCCTGAACGTG GCT GGC	462	
Bla _DHA-1_, Bla _DHA-2_	AACTTTCACAGGTGTGCTGGGT CCGTACGCATACTGG CTT TGC	405	
Bla _ACC_	AACAGCCTCAGCAGCCGGTTA TTCGCCGCAATCATC CCT AGC	346	
Bla _MIR-1_, Bla _ACT-1_	TCGGTAAAGCCGATGTTGCGG CTTCCACTGCGGCTGCCAGTT	302	
Bla _FOX-1_, Bla _FOX-5 B_	AACATGGGGTATCAGGGAGATG CAAAGCGCGTAACCGGATTGG	190	
Bla _SHV_	ATGCGTTATATTCGCCTGTG TGCTTTGTTAT CGGGCCAA	747	[43]
Bla _TEM_	TCGCCGCATACACTATTCTCG AATGA ACGCTCACCGGCTCCA GATTTAT	445	
Bla _CTXM_	ATGTGCAGYACCAGTAARGTK ATGGC TGGGTRAARTARGTSACCAGAAYCAGCGG	593	

**Table 6:** Preparations of the PCR reaction.

PCR Reaction Mixture	Reaction Volume	
	Conventional PCR	Multiplex PCR
2x Taq Master Mix	5 μL	20 μL
PCR grade water	2 μL	3 μL
Forward primer	1 μL	6 μL
Reverse primer	1 μL	6 μL
Template DNA	1 μL	5 μL
Total	10 μL	40 μL

## 5. Conclusions

This study revealed the phenotypic and genotypic link of ESBL-producing *Enterobacteriaceae* isolated from humans, cattle, and poultry, suggesting that cattle and poultry could be a potential reservoir host of those bacteria with a risk of infecting the human population. Our results emphasize continuous monitoring and obtaining more samples to investigate the genetic relationship between animal and human bacterial isolates. Moreover, advanced molecular epidemiological studies, such as whole-genome sequencing, are required in order to better understand the zoonotic potential of those bacteria at the human–animal interface. Additionally, the dissemination of AMR should be checked thoroughly by introducing surveillance programs on hospital sites and animal and poultry production farms. Besides this, antibiotics should be appropriately used in Egypt’s veterinary and medical industries.

## Figures and Tables

**Table 1:** The prevalence of ESBL-producing Enterobacteriaceae in humans, cattle, and poultry farms.

Sources of samples	Samples	No. of samples	*E. coli*		*Klebsiella*		Total	
			Positive	%	Positive	%	Positive	%
Farm workers samples	Fecal samples	20	4	20.0	2	10.0	6	30.0
	Total	20	4	20.0	2	10.0	6	30.0
Cattle farm samples	Calves rectal swabs	17	2	11.76	1	5.88	3	17.65
	Cows rectal swabs	17	2	11.76	2	11.76	4	23.53
	Milk	14	2	14.28	1	7.14	3	21.43
	Milking machine swabs	8	1	12.5	1	12.5	2	25.0
	Ration	2	0	0.00	0	0.00	0	0.00
	Water	2	0	0.00	0	0.00	0	0.00
	Total	60	7	11.67	5	8.33	12	20.0
Poultry farms samples	Cloacal swabs	45	5	11.11	3	6.67	8	17.78
	Ration	5	1	20.0	2	40.0	3	60.0
	Water	5	1	20.0	1	20.0	2	40.0
	Litter	5	1	20.0	1	20.0	2	40.0
	Total	60	8	13.33	7	11.67	15	25.0

**Table 2:** Results of the antimicrobial susceptibility testing of ESBL-producing *Enterobacteriaceae* among humans, cattle, and poultry.

Antibiotics	*E. coli*				*Klebsiella*				Total			
	Type	Number	Resistant	%	Type	Number	Resistant	%	Type	Number	Resistant	%
**Cefotaxime**	Human	4	3	75.0	Human	2	1	50.0	Human	6	4	66.67
	Cattle	7	5	71.43	Cattle	5	5	100.0	Cattle	12	10	83.33
	Poultry	8	7	87.5	Poultry	7	7	100.0	Poultry	15	14	93.33
									**Total**	**28/33**		**84.85**
**Ceftazidime**	Human	4	2	50.0	Human	2	2	100.0	Human	6	4	66.67
	Cattle	7	7	100.0	Cattle	5	4	80.0	Cattle	12	11	91.67
	Poultry	8	7	75.0	Poultry	7	6	85.71	Poultry	15	13	80.0
									**Total**	**28/33**		**84.85**
**Amoxyclavulanic**	Human	4	1	25.0	Human	2	0	0.00	Human	6	1	16.67
	Cattle	7	1	14.28	Cattle	5	1	20.00	Cattle	12	2	16.67
	Poultry	8	2	25.0	Poultry	7	2	28.57	Poultry	15	4	26.67
									**Total**	**7/33**		**21.21**
**Levofloxacin**	Human	4	2	50.0	Human	2	2	100.0	Human	6	4	66.67
	Cattle	7	2	28.57	Cattle	5	2	40.0	Cattle	12	4	33.33
	Poultry	8	6	75.0	Poultry	7	6	85.7	Poultry	15	12	80.0
									**Total**	**20/33**		**60.61**
**Imipenim**	Human	4	1	25.0	Human	2	0	0.00	Human	6	1	16.67
	Cattle	7	0	0.00	Cattle	5	1	20.0	Cattle	12	1	8.33
	Poultry	8	1	12.5	Poultry	7	0	0.00	Poultry	15	1	6.67
									**Total**	**3/33**		**9.1**
**Cefepime**	Human	4	3	75.0	Human	2	2	100.0	Human	6	5	83.33
	Cattle	7	4	57.14	Cattle	5	3	60.0	Cattle	12	7	58.33
	Poultry	8	6	75.0	Poultry	7	5	71.43	Poultry	15	11	73.33
									**Total**	**23/33**		**69.7**

**Table 3:** Distribution of ESBL-encoding genes in the isolated *E. coli* and *Klebsiella,* as well as their antimicrobial resistance phenotype.

Isolates	Origin	Resistance Gene Pattern				Antimicrobial Resistance
		*bla*_CTX-M_	*bla*_SHV_	*bla_TEM_*	*Amp*C	
*Ec1*	Human	+	+	+		CTX, CAZ, AMC, LEVO, IPM
*Ec2*	Human		+	+		CTX, CAZ, LEVO, FEP
*Ec3*	Human	+	+	+		CTX, FEP
*Ec4*	Human		+			FEP
*Ec5*	Cattle		+			CTX, CAZ, LEVO, FEP
*Ec6*	Cattle	+		+		CTX, CAZ, LEVO, FEP
*Ec7*	Cattle	+	+	+		CTX, CAZ, FEP
*Ec8*	Cattle		+			CTX, CAZ, FEP
*Ec9*	Cattle		+	+		CTX, CAZ,
*Ec10*	Cattle	+	+	+	+ (*bla*_Fox_ & *bla*_DHA_)	CAZ, AMC
*Ec11*	Cattle		+	+		CAZ
*Ec12*	Poultry		+	+		CTX, LEVO, IPM, FEP
*Ec13*	Poultry	+	+	+		CTX, CAZ, AMC, LEVO, FEP
*Ec14*	Poultry	+		+	+ (*bla*_Fox_ & *bla*_DHA_)	CTX, CAZ, AMC, FEP
*Ec15*	Poultry	+	+	+		CTX, CAZ, LEVO, FEP
*Ec16*	Poultry	+	+	+		CTX, CAZ, LEVO, FEP
*Ec17*	Poultry	+	+	+		CTX, CAZ, LEVO, FEP
*Ec18*	Poultry		+	+		CTX, CAZ
*Ec19*	Poultry	+	+			CAZ, LEVO
Kl1	Human	+	+	+		CTX, CAZ, LEVO, FEP
Kl2	Human		+	+		CAZ, LEVO, FEP
Kl3	Cattle	+	+	+		CTX, CAZ
Kl4	Cattle	+	+	+		CTX, CAZ, LEVO, FEP
Kl5	Cattle		+	+		CTX, CAZ, LEVO, FEP
Kl6	Cattle	+	+	+	+ (*bla*_Fox_ & *bla*_DHA_)	CTX, CAZ, AMC
Kl7	Cattle	+	+	+		CTX, IPM, FEP
Kl8	Poultry	+	+	+	+ (*bla*_ACC_)	CTX, CAZ, AMC, LEVO, FEP
Kl9	Poultry	+	+	+	+ (*bla*_ACC_)	CTX, CAZ, AMC, LEVO, FEP
Kl10	Poultry	+	+	+		CTX, CAZ
Kl11	Poultry	+	+	+		CTX, CAZ, LEVO, FEP
Kl12	Poultry	+	+	+		CTX, CAZ, LEVO, FEP
Kl13	Poultry	+	+	+		CTX, CAZ, LEVO
Kl14	Poultry	+	+	+		CTX, LEVO, FEP
**Total**	**NO.**	23	31	29	5	
	**%**	69.70	93.94	87.88	15.15	

CTX, Cefotaxime; CAZ, Ceftazidime; FEP, Cefipime; LEVO, Levofloxacin; AMC, Amoxyclavulanic acid.

**Table 4:** Selected *E. coli* and *Klebsiella* isolates for genetic analysis.

Isolates	ID	Origin	GenBank Accession No.
*Klebsiella*	KHF	Human	MZ461491
*Klebsiella*	KCF	Cattle	MZ461492
*Klebsiella*	KPF	Poultry	MZ461493
*E. coli*	EHF	Human	MZ461494
*E. coli*	ECF	Cattle	MZ461495
*E. coli*	EPF	Poultry	MZ461496

## Data Availability

The data are available upon request from the corresponding author.
